# Exploring patient-provider interactions and the health system’s responsiveness to street-connected children and youth in Kenya: a qualitative study

**DOI:** 10.1186/s12913-021-06376-6

**Published:** 2021-04-20

**Authors:** Embleton Lonnie, Shah Pooja, Gayapersad Allison, Kiptui Reuben, Ayuku David, Wachira Juddy, Apondi Edith, Braitstein Paula

**Affiliations:** 1 Dalla Lana School of Public Health, University of Toronto, Health Sciences Building, 155 College Street, 6th Floor, Toronto, ON M5T 3M7 Canada; 2 Institute of Medical Sciences, Faculty of Medicine, University of Toronto, 1 Kings College Circle Room 2374, Toronto, ON M5S 1A8 Canada; 3 London School of Hygiene & Tropical Medicine, Keppel St, Bloomsbury, London, WC1E 7HT UK; 4 Academic Model Providing Access to Healthcare (AMPATH), P.O. Box 4606-30100, Eldoret, Kenya; 5 Department of Behavioural Science, Moi University, College of Health Sciences, P.O. Box 4606-30100, Eldoret, Kenya; 6 Moi Teaching and Referral Hospital, Eldoret, Kenya; 7 Moi University, College of Health Sciences, School of Medicine, P.O. Box 4606-30100, Eldoret, Kenya

**Keywords:** Street children, Kenya, Health systems responsiveness, Patient-provider interactions

## Abstract

**Background:**

In Kenya, street-connected children and youth (SCY) have poor health outcomes and die prematurely due to preventable causes. This suggests they are not accessing or receiving adequately responsive healthcare to prevent morbidity and mortality. We sought to gain insight into the health systems responsiveness to SCY in Kenya through an in-depth exploration of SCY’s and healthcare provider’s reflections on their interactions with each other.

**Methods:**

This qualitative study was conducted across 5 counties in western Kenya between May 2017 and September 2018 using multiple methods to explore and describe the public perceptions of, and proposed and existing responses to, the phenomenon of SCY in Kenya. The present analysis focuses on a subset of data from focus group discussions and in-depth interviews concerning the delivery of healthcare to SCY, interactions between SCY and providers, and SCY’s experiences in the health system. We conducted a thematic analysis situated in a conceptual framework for health systems responsiveness.

**Results:**

Through three themes, context, negative patient-provider interactions, and positive patient-provider interactions, we identified factors that shape health systems responsiveness to SCY in Kenya**.** Economic factors influenced and limited SCY’s interactions with the health system and shaped their experiences of dignity, quality of basic amenities, choice of provider, and prompt attention. The stigmatization and discrimination of SCY, a sociological process shaped by the social-cultural context in Kenya, resulted in experiences of indignity and a lack of prompt attention when interacting with the health system. Patient-provider interactions were highly influenced by healthcare providers’ adverse personal emotions and attitudes towards SCY, resulting in negative interactions and a lack of health systems responsiveness.

**Conclusions:**

This study suggests that the health system in Kenya is inadequately responsive to SCY. Increasing public health expenditures and expanding universal health coverage may begin to address economic factors, such as the inability to pay for care, which influence SCY’s experiences of choice of provider, prompt attention, and dignity. The deeply embedded adverse emotional responses expressed by providers about SCY, associated with the socially constructed stigmatization of this population, need to be addressed to improve patient-provider interactions.

**Supplementary Information:**

The online version contains supplementary material available at 10.1186/s12913-021-06376-6.

## Background

Homeless populations in high-income countries face significant barriers to accessing healthcare [1, 2]. In low- and middle-income countries, such as Kenya, some evidence suggests that street-connected children and youth (SCY), for whom the streets plays a central role in their everyday lives and social identities [3], also encounter barriers to accessing care due to distrust in the quality of care, cost, time away from income generation, and stigmatization by healthcare providers, all of which may contribute to the significant morbidities and health disparities experienced by this population [4]. In Kenya, SCY have poor health outcomes including a high prevalence of HIV and sexually transmitted infections [5–8], unfavourable reproductive health outcomes [9], growth and development disparities [10, 11], respiratory problems [12], and mental health and substance use issues [13–16]. SCY die prematurely of preventable causes [17, 18], suggesting that they are not accessing or receiving adequately responsive healthcare. It is likely that SCY in Kenya endure multiple barriers to care due to their social and economic marginalization and stigmatization [8, 19–21]. These barriers intersect with the structural and other drivers of children and youth to the street, notably poverty, family conflict, and child abuse, that create numerous social and health inequities [20, 22, 23].

In Kenya, healthcare utilization is tied to socioeconomic status. Substantial inequities exist in healthcare utilization, with vulnerable and low-income populations reporting poorer health status and lower utilization of health services [24]. Kenya has made a pledge to realise universal health coverage by 2022 with the goal of reducing these inequities, but research demonstrates significant gaps remain and the poorest populations are still being left behind [25, 26]. Public expenditure on healthcare in Kenya is limited [26], yet research suggests that higher public health expenditures is associated with health system responsiveness for the poorest individuals [27]. Further, health system responsiveness may be lower among individuals of low socio-economic status [28]. Many SCY in Kenya migrate to the streets due to extreme poverty, have low levels of education and minimal daily earnings, [14, 19, 20], thus may be at high risk of experiencing poor health system responsiveness.

The World Health Organization (WHO) outlines that responsiveness in the context of health systems is an outcome that can be attained when institutions and institutional relationships are devised to be cognizant of and respond to the expectations of individuals [29]. A review of the literature on health system responsiveness by Mirzoev and Kane (2017), identified the central importance of interactions between users and the health system and concluded that ‘*health systems responsiveness is best studied as an experience of an interaction which people have during their encounter with a health system*’. Mirzoev and Kane (2017) recognised several gaps in existing frameworks and concluded that they did not adequately capture people’s interactions with and expectations of providers and health systems, nor did they sufficiently address contextual factors that influence these interactions. As a result, they proposed a comprehensive health systems responsiveness framework, building on the seven elements (dignity, autonomy, confidentiality, prompt attention, quality of amenities, access to social support networks and choice of service provider) of the WHO’s widely used health systems responsiveness framework [29, 30].

In their conceptual framework on health systems responsiveness, Mirzoev & Kane (2017) locate people’s interactions and experiences with their providers and the health system in the context of the seven elements at the core of their framework and add an eighth element of trust. Their framework recognises that various determinants influence these interactions from both the health system and the people’s sides [30]. These determinants may include, but are not limited to, people’s expectations of their health system, providers and the health system’s response to people’s expectations, providers perceptions of users and attitudes, organization and management of the health system, and historical, political, cultural and socioeconomic contextual factors that influence and shape the interactions between people and the health system [30–33].

Given the significant preventable morbidities and mortality experienced by SCY in Kenya [6–8, 10, 13, 14, 16, 17], which may suggest a lack of health system responsiveness to this population, Mirzoev & Kane’s conceptual framework can be used to gain insight into how the health system is or is not responsive to this population. It has been established that people’s reflections on their experiences of interacting with their health system is a proxy for measuring a health system’s responsiveness [30]. Therefore, we sought to gain insight into the health system’s responsiveness to SCY in our setting through an in-depth exploration of SCY’s and healthcare providers reflections on their interactions with each other. Specifically, we analyse these patient-provider interactions, while taking into account how socially constructed perceptions of SCY and the emotions of healthcare providers influence patient-provider interactions, and how broader contextual factors shape the health system responsiveness to SCY in Kenya.

## Methods

### Study design

The present qualitative analysis uses a subset of data from focus group discussions (FGDs) and in-depth interviews (IDIs) that focused on the delivery of healthcare to SCY, interactions between SCY and providers, and SCY’s experiences in the health system. This qualitative study, which sought to broadly explore and describe the different public perceptions of SCY from various social actors, was conducted between May 2017 and September 2018. In-depth details regarding the study design have been previously reported [20, 21].

### Study setting

This study was conducted in Eldoret, Kitale, Nakuru, Kisumu, and Bungoma in Western Kenya, due to the large numbers of SCY who exist in these locations [7, 34]. The research team was based in Eldoret, in Uasin Gishu county, which was the primary study site. Additional details regarding the study sites have been previously reported [20, 21].

### Study participants, recruitment, and enrolment

Table 1 presents information on study participants, locations, and the types of interviews conducted. In-depth details on the study participants, recruitment, and enrolment have been previously reported [20, 21]. In brief, healthcare providers were purposively contacted through our established networks and contacts. Physicians, clinicians, nurses, social workers, and HIV testing and counselling practitioners were interviewed in order to gather opinions from a wide range of professionals working in healthcare who may interact with SCY in the health system. Healthcare participants were consented and interviewed at the referral hospital in Uasin Gishu or Moi University. SCY aged 15–24 years were invited to voluntarily participate after extensive outreach and study sensitization, which occurred in the locations they are known to live and work in each study site. For SCY, the process of enrolment and consent took place at the Rafiki Centre for Excellence in Adolescent Health at MTRH in Uasin Gishu county, and on the streets in locations where SCY congregate in the other study locations.

**Table 1:** Participants, interviews, and location

Social actors	# of interviews	Location	Sex of interviewees
Community leaders	4	Uasin Gishu	4 Men
County Children’s Coordinators	1	Uasin Gishu	1 Man
Police Officers	6	Uasin Gishu, Nakuru Trans-Nzoia, Kisumu, Bungoma	3 Women, 3 Men
Children’s Officer(s)	6	Uasin Gishu, Nakuru Trans-Nzoia, Kisumu, Bungoma	2 Women, 4 Men
Vendors	2	Uasin Gishu	1 Woman, 1 Man
General Community	3	Uasin Gishu	1 Woman, 2 Men
CBO / Stakeholders & SCY Advocates	6	Uasin Gishu	2 Women, 4 Men
Peer Navigators	2	Uasin Gishu	1 Woman, 1 Man
Parents of Street children	1	Uasin Gishu	1 Woman
Former Street-connected youth	3	Uasin Gishu	2 Women, 1 Man
Street-connected youth	7	Uasin Gishu, Kisumu, Trans-Nzoia	5 Women, 2 Men
**Total In-depth Interviews**	**41**		**18 women, 23 men**
AMPATH Clinicians	FGD	Uasin Gishu	2 women, 3 men
AMPATH Nurses, Social Work, Counsellors	FGD	Uasin Gishu	4 Women, 2 Men
MTRH Clinicians	FGD	Uasin Gishu	6 Men
MTRH Nurses	FGD	Uasin Gishu	6 Women
SCY Males FGD	FGD	Uasin Gishu	12 men
SCY Females FGD	FGD	Uasin Gishu	12 women
Mixed gender SCY Nakuru	FGD	Nakuru	6 Young women, 6 Young men
**Total Number of FGDs**	7		**30 women, 29 men**

### Ethics and consent

This study received approval from University of Toronto Research Ethics and the Moi University, MTRH Institutional Research Ethics Committee. A waiver of parental consent was obtained for minors, and written informed consent or assent was obtained from all participants in the study. Participants were made aware that their interviews would be audio-recorded, and that if at any time during the interview they were uncomfortable and no longer wished to continue, they could leave freely without any repercussions. In total, nine participants declined to be audio-recorded, but gave the interviewer permission to take notes. Participants were compensated for their time. SCY were provided with 200 Kenyan Shillings (Ksh) (~USD 2), and all other participants were provided with 1000 Ksh (~USD 10). Participants in the focus groups were provided with refreshments and travel reimbursement.

### Data generation

In-depth details about data generation have been reported elsewhere [20]. In brief, IDIs and FGDs were conducted in either English, Swahili or a mixture of the two languages by a team of trained interviewers. On average, IDIs were 40 min long, and FGDs were 1.5 h long. Using a pre-tested semi-structured interview guide, participants were asked about their perceptions of SCY in general, their experiences of interactions with SCY, and their thoughts on the needs of SCY. Healthcare providers were asked additional questions relating to the provision of healthcare of these individuals. SCY were interviewed using a separate guide which questioned them about their interactions with and experiences of the general community, their perceptions of their own needs, their ability to access healthcare and other social services, and how they are treated when coming for healthcare. The interview guides are available in Additional File 1.

### Qualitative data analysis

The analytic process began with comprehensive reading of translated and transcribed data. In a collaborative and iterative process, we developed a codebook that captured key components of Mirzoev & Kane’s conceptual framework for health systems responsiveness including interactions between SCY and healthcare providers, positive experiences, negative experiences, political, economic, and social-cultural contextual determinants, the health system and facilities, and healthcare provider attitudes (actual and those perceived by SCY). Based on this initially developed codebook, we test-coded transcripts using thematic analysis [35]. In subsequent analytic meetings to review and discuss the functionality of the codebook, we identified that participants consistently discussed their emotions when recounting their interactions (healthcare providers and SCY) and the experiences of those interactions. We therefore amended our codebook to capture emotions expressed by SCY and healthcare providers. During our analysis we categorized these into ‘positive’ and ‘negative’ emotions. Positive emotions included feelings such as happiness, empathy, compassion, and being grateful. Negative emotions included feelings such as fear, sadness, pity, shame, and ambivalence. The final codebook was evaluated for its comprehensiveness and reliability by four authors (PS, LE, AG, RK) test-coding a series of transcripts and comparing them for consistency. This final thematic analysis was carried out by the same four authors and analytic notes and annotations from each author were documented and discussed in meetings. After all transcripts were coded, a series of analytic meetings were conducted to discuss how the data mapped onto core concepts of health systems responsiveness and to analytically explore how the interactions described by SCY and healthcare providers were shaped by perceptions/attitudes, emotions, and contextual factors. We used NVivo software version 11 for coding and analysis.

## Findings

We conducted 41 IDIs and 7 FGDs with a total of 100 participants, of whom 48 were women and 52 were men. The median age of street-involved participants was 16 years, and of community participants, 42 years.

Our analysis explores the interactions between SCY, healthcare providers and the health system in Kenya, and identifies what factors shape both the health system’s and SCY’s sides of these interactions. First, we identify and examine how contextual-level determinants shape SCY’s interactions with the health system. Second, we highlight how perceptions and emotions of healthcare providers shape these interactions leading to ‘negative’ or ‘positive’ patient-provider interactions, thereby influencing health system responsiveness. Thus, we present our analysis of health system responsiveness to SCY in this context through these three major themes: context, positive patient-provider interactions, and negative patient-provider interactions.

### Contextual-level determinants of health systems responsiveness

Historical, social, cultural, political, and economic contextual factors influence people’s interactions with their health systems, and shape the health system’s ability to adequately respond to people’s expectations [30]. We generated two sub-themes covering the economic and socio-cultural context that influence SCY’s interactions with their health system.

### Economic context

Economic factors influence and limit SCY’s interactions with the health system and our analysis reveals how economic context shapes SCY’s experiences of dignity, quality of basic amenities, choice of provider, and prompt attention. Public healthcare facilities in Kenya offer a degree of universal health coverage for maternal, neonatal, and child health services. Fee-for-service remains for other out-patient and in-patient care, laboratory and radiology diagnostic services, and pharmaceuticals. SCY consistently expressed that they may not be treated due to a ‘lack of money’ when seeking healthcare. One participant recounted a case where the cost of healthcare resulted in a street-connected boy’s death:

When you go to the hospital, they will need money, no hospital is free because you will have to buy drugs. I brought a boy here who was vomiting blood, and scanning and x-ray had to be done and it would cost 15,000 shillings [~ $145 USD] and we can’t afford that, so he just suffered until he died. So, the main problem is lack of money.

(Former street-connected young man)

Without money, SCY reflected that they were treated without dignity in the health system and without respect by healthcare staff as expressed by one young man:

No, I refuse because when you go there, they look at you like trash. If you have no money, they will just look at you.

(Street-connected young man)

The lack of financial resources to pay for services and care out-of-pocket limits SCY’s choice of provider. Kenya has private and public healthcare facilities where citizens enrolled in the National Health Insurance Fund can receive care if the facility is accredited. As one group of healthcare provider participants discussed, SCY are limited to accessing government facilities as they are uninsured:

Government facilities … It’s easily accessible, they don’t have to undergo too much security for them to be treated, they will be treated without being asked first how they will pay. They don’t have money nor an insurance cover, where else can they go to? They can’t go to Mediheal [private hospital].

(FGD, Nurses, Counsellors, and Social workers)

SCY confirmed a limited choice of provider due to economic constraints. In addition to being limited by which clinics they access; they are also limited in their ability to get follow-up or specialised care beyond basic primary care and will go where care is free, as explained by one street-connected young woman:

I went to District hospital when my son was sick. He got better, then we came home. I also went to the prison clinic. They gave me a card, I have it at home. They helped me get a card, so I didn’t have to pay. I can access services at the prison clinic easily. Everywhere else they ask for money.

(Street-connected young woman)

The economic and resource constraints in Kenya also impact the ability of the health system to be responsive to SCY’s additional needs, and ensure they have sufficient basic amenities as an in-patient as explained by a nurse:

They are just like any other patient, but they need special needs like the one that came to my ward had wounds and when you serve food, he was not getting enough, that’s why I am saying they need special attention.

(FGD, Nurses)

The ability to pay for health services influenced SCY’s experiences of prompt attention when seeking healthcare as described by one street-connected young woman:

We don’t pay^1^ so they are not in a hurry to treat us; they want people who come with money.

(FGD, Street-connected young women)

SCY reflected upon how their ability to pay intersected with aspects of their social identity and appearance, thus influencing their experience of prompt attention and dignity in their interactions with the health system and providers.

They feel like you are disturbing them, the others are clean, and you don’t have money. You may find a good doctor who will treat you well or a bad one who will chase you away.

(FGD, Street-connected young women)

SCY experience substantial stigmatization and discrimination due to their identity, appearance, and stereotypes associated with this marginalized population [21].

### Social-cultural context

Stigmatization and discrimination of SCY is a sociological process greatly influenced by the social-cultural context in the region, including the media, religion, and cultural conceptions of childhood [21]. It results in experiences of indignity and a lack of prompt attention when interacting with the health system. Beyond SCY and providers, government officials recognized that SCY experience discrimination at hospitals when interacting with the health system:

They are treated nicely sometimes for those ones who know the processes [to receive a waiver for care], but because most of them don’t know, they always express disgust of the services and generally discrimination that they receive in the hospitals. We cannot refuse that sometimes they are discriminated and looked down upon, but those who know their ways around they get services without being charged.

(Children’s Officer)

Due to their socially stigmatized identity as ‘*chokoraa*’ (garbage pickers), physical appearance, and use of substances [21], several SCY across cities recounted difficulties when interacting with providers in the health system, which also resulted in experiences of indignity and not receiving prompt care as described by two SCY:

I have to go very early. Others get treated first as I am a *chokoraa* [garbage picker], so it’s not easy to get treated. I don’t get treated as I should be. They rush through it just so I will leave. It makes me feel bad.

(Street-connected young man)

When I go with you while we are both sick, at the queue I will not be attended to first, I will just stay there. You know we are usually stubborn on the streets but if I am not, I will be treated. Even if you are badly injured you will not be treated first until we protest. They usually tell us to let them attend to people first, so I don’t know if they think we are not humans because they usually tell us to wait.

(FGD, Street-connected young women)

SCY experiencing indignity and a lack of prompt attention as a result of their socially constructed stigmatized identity [21] when interacting with the health system was confirmed by a Peer Navigator, who is specially trained to assist SCY to navigate the health system [5] and receive care:

They are treated differently because of the attitudes people have towards them and they are dirty. They can go there badly injured, but there is no emergency care or treatment. Like the other day at [hospital redacted] I had taken a client who had a badly injured arm, he had stayed with it for long, so it had pus. But the people at the records kept referring us to different rooms and I begged them to assist him because he was suffering; it’s not that they didn’t want to help, but we had to go to a social worker first. When you go to a social worker you would still be sent to room 14 where you would be sent to another room. The social worker will try to divert the situation because we have to start at the district [hospital] first. So, you have to come with a letter from district, that is a long process if it’s an emergency. They delay treatment.

(Peer Navigator)

As the Peer Navigator stated, SCY’s treatment is shaped by the attitudes people have towards them, which are formed as a result of widely held social-cultural perceptions and stigmatization of SCY [21] that we theorise directly influences patient-provider interactions.

### Patient-provider interactions

Our analysis led us to theorise that patient-provider interactions are influenced by personal emotions that are the result of socially constructed perceptions about SCY in this context. We show that providers perceptions of and emotional responses to SCY are the result of widely held socially constructed views about this population, which associates them with the ‘evils in society’, theft/robbery, and violence, and leads to their stigmatization and discrimination [21]. We theorise that these perceptions lead to ‘positive’ or ‘negative’ emotional responses in the context of patient-provider interactions, resulting in SCY’S ‘positive’ or ‘negative’ interactions with providers and the health system. Table 2 shows the emotions expressed by healthcare providers with respect to SCY and quantifies the number of times providers reference specific emotions. Table 3 shows the emotions expressed by SCY with respect to how they feel and are treated by the public at large. The emotions captured in these tables are not solely in the context of interactions in the health system, nonetheless we theorise that these emotions shape and influence the patient-provider interactions as they are held by providers and patients.

**Table 2:** Emotions expressed by providers in the health system with respect to street-connected children and youth in Kenya

Emotions / Attitudes	Definition of the emotion	# of times language used	Example of a quote from a healthcare provider
**Negative Emotions**			
Fear	An unpleasant emotion caused by the threat of danger, pain, or harm	24	Sometimes you don’t pity them because they can bully you (laughs) if no one is around or you cross where they stay and it’s dark, they can really torture you or steal from you. Sometimes I fear them. (Nurses)
Pity	A feeling of sadness or sympathy for someone else’s unhappiness or difficult situation ‘I acknowledge your suffering’	21	Being on the streets means that they are needy, homeless and they don’t have basic needs nor anyone caring about their health. They don’t have a place to sleep and generally that is a sorry state. (Nurses)
Ambivalence	The state of having mixed feelings or contradictory ideas about something or someone	6	I have mixed feelings; I feel sad and I also feel nothing for them. It’s sad to see them there but another part of me doesn’t. (Clinicians)
Annoyed	Slightly angry; irritated.	5	Because they are bothersome, they really want to interfere with what you are doing. (Nurses, Counsellors, Social workers)
Sad	Feeling or showing sorrow; unhappy.	3	They are suffering during the cold and rainy season; you have to feel sad because we are humans. (Clinicians)
Shame	A painful feeling of humiliation or distress caused by the consciousness of wrong or foolish behaviours.	3	Like him, I feel pity for some but for others I don’t because they come to the streets looking for something. For ladies I don’t feel any pity for them because sometimes when you see what they are doing you feel so ashamed. When you pass and see the boda boda men pushing with them you feel so ashamed, they should even try to work as house helps. (Clinicians)
Disappointment	Sadness or displeasure caused by the non-fulfilment of one’s hopes or expectations	1	If I can add, I feel disappointed because ideally, they are not supposed to be on the streets, if we do a follow up everyone has a family associated with them and they are able to provide not necessarily all their needs. Because of failed values in the society, we have now abandoned them to fend for themselves and God will take care of the rest. (Clinicians)
Dislike	To feel distaste for	1	I don’t like the big ones. (Clinicians)
Dread	An emotion experienced in anticipation of some specific pain or danger (usually accompanied by a desire to flee or fight)	1	I know of one who used to be our patient, I don’t know if I can say it was mental illness or being in the streets. We met at the market and he forced me to buy his bananas, yet they were rotten (laughs) he knew me because we had interacted once in the ward. Sometimes we meet and he blocks the way and people just pass by, no one helps. When he forced me to buy the bananas, I just gave him 100 shillings and left. M: What would happen if you didn’t buy from him? R: I think he just wanted the money, robbery without violence (laughs) I still dread meeting him. Most of them when we meet, I just avoid them and go to another street. (Nurses)
Frustrated	Feeling or expressing distress and annoyance resulting from an inability to change or achieve something	1	I feel like there is a big problem, it’s a wasted energy because these are people who can look for employment and do something to build their lives and also the economy at large because on the streets most of them engage in illegal things like abusing drugs and torturing people. You can imagine them channeling that energy into something constructive, even for themselves, it would be a better nation. Wasted life, wasted manpower. (Nurses)
Hostile	Showing or feeling opposition or dislike; unfriendly. Or [anger feeling or showing strong annoyance, displeasure, or hostility; full of anger.]	1	When you talk to them especially at the parking lot in Zion mall they will tell you a lot of stories on why they are on the streets, when you ask them if they would go to children’s homes or they will run away, the answers they will give you will make you harsh again. (Clinicians)
**Positive Emotions**			
Sympathy	The feeling that you care about and are sorry about someone else’s trouble, grief, misfortune, etc. ‘I care about your suffering’	12	I sympathize with those who found themselves on the streets and don’t even know their families and I may even give them some cash because they have nowhere to go or something to eat. (Clinicians)
Happy	Feeling or showing pleasure or contentment.	5	[How do you normally feel when they come for HIV testing?] … It’s a good feeling, at least they have some positivity in themselves, they care about themselves in as much as they are on the streets, they have to be safe. You have to talk more to them so that they can have knowledge. (Nurses, Counsellors, Social workers)
Empathy	The feeling that you understand and share another person’s experiences and emotions; the ability to share someone else’s feelings	4	I feel like they are my brothers because I once lived with them and I love them. If I see someone beating them like the askaris I usually feel bad because they are harassing them. If I meet them, I usually hug them no matter how smartly dressed I am and people always look at me wondering, they don’t know what makes me close with them. (Peer Navigator)
Compassion	Concern for the sufferings or misfortunes of others with a desire to alleviate it	3	We need to make them feel like they are humans too, don’t rehabilitate me yet you hate me [so much] that you can’t come close to me. Love your neighbor as you love yourself …. They also need love, if you rehabilitate me yet you hate me it won’t add any value. (Clinicians)

**Table 3:** Emotions expressed by street-connected children and youth in Kenya with respect to how they feel and are treated in the community

Emotions / Attitudes	Definition of the emotion	# of times language used	Example of a quote from SCY community
**Negative emotions**			
Alienated	Experiencing or inducing feelings of isolation or estrangement	7	You feel bad, you ask why they isolate you, yet you are one of them? But I know we are different because they have it [all] while I don’t.
Sad	Feeling or showing sorrow; unhappy.	7	We feel very bad and often ask God what did I do to deserve this!
Pity	A feeling of sadness or sympathy for someone else’s unhappiness or difficult situation ‘I acknowledge your suffering’	1	I pity them [SCY] and I pray to God to assist them.
Stressed	Experiencing mental or emotional strain or tension	1	I am usually stressed at the cell. When they arrest me, I usually cry and ask God for help.
Ambivalence	The state of having mixed feelings or contradictory ideas about something or someone	1	Some [people] care about me and some don’t
**Positive emotions**			
Happy	Feeling or showing pleasure or contentment.	3	There are some who take us well. People at OSCAR and Berur who help us. I feel good because if it weren’t for them, we would be suffering like not being able to get treatment.
Grateful	Feeling or showing an appreciation for something done or received	2	I can say this.... there are people in town who love us. I can tell them about my problems at home and they get money from their pockets to help me. But there are those who will say no because we are street children. I do my own work and I am grateful for the little I get.

### Positive patient-provider interactions

Healthcare providers frequently expressed sympathy with respect to the plight of SCY, and in a few cases compassion and empathy (Table 2). One street-connected young man discussed that clinicians were one group of people that treated them well regardless of their social identity and appearance:

Everyone, all types of people including the police, think we are thieves. But not doctors, they treat us okay. There are also people that come and teach us about things like cleanliness. Matatu [public bus] drivers ask us for money even before we enter the car. Other passengers want to move away from us because we smell. But doctors treat us well.

(Street-connected young man)

A few SCY discussed that when ill, they are treated well by providers and are able to receive care:

The health providers treat us well as they attend to us when we are ill. You see like my husband here we took him, and he got treated on this leg that has this deep cut.

(FGD, Street-connected young men and women)

Overall, there were few reflections of positive interactions between SCY and providers within the health system.

### Negative patient-provider interactions

Adverse emotions prevailed among healthcare providers, with participants most frequently discussing that they felt fear, pity, ambivalence, and annoyance with respect to SCY (Table 2). As one clinician explains, people in the clinic feel fearful when interacting with them:

People really get scared by the way (laughs), even the children, because they look dirty and rowdy. They walk carelessly, some sniffing glue. Most of us are not used to that stench of glue and you can even get a headache.

(FGD, Clinicians)

The emotional response of fear may lead to a lack of trust between providers and patients as one provider describes:

I ask myself why can’t they just shower and be clean like everyone else? Because they come here [to the clinic] with a bad stench, they can’t even bath when sick? You feel that they are so dirty. Most of the times you feel insecure because they can take off with your phone, so you have to be alert always.

(FGD, Nurses, Counsellors, Social Workers)

These emotional responses are associated with the widely held perception that SCY are predominantly responsible for crimes, as revealed in a clinician’s reflection:

I used to work in the emergency unit at [hospital redacted], they mug people in town, serious mugging. They can even hack you and take everything you have. In some cases, ladies are raped. People get mugged mostly late in the evening.

(FGD, Clinicians)

By and large, providers did not report having positive interactions with SCY within or outside of the health system:

For me I have never had any positive experience with them … When I meet them, I really don’t want to interact with them because of my previous experiences and their behaviors, some of them abuse drugs so I just try to avoid them irrespective of the age and gender.

(FGD, Clinicians)

Despite these adverse emotional responses and disdain for interacting with SCY, providers consistently expressed that SCY have a right to healthcare as affirmed by one nurse ‘*they are human beings and, in the constitution, everyone has a right to healthcare*’. Providers’ reflections demonstrate contradictions between their moral obligation to provide care as a professional and their apprehension about interacting with SCY. More often than not, SCY described negative interactions with healthcare providers when seeking care. SCY recognized that there were treated differently due to their appearance and social identity, which is highly stigmatized, leading to experiencing a lack of dignity as explained by a participant:

They treat us with contempt because we are dirty but if you are clean, you will be treated. They have preferences.

(FGD, Street-connected young men)

SCY predominantly expressed feeling alienated and sad as a result of how they are treated by the community (Table 3). In the context of healthcare, SCY expressed negative emotions associated with their interactions with healthcare providers as a result of enduring experiences of indignity and a lack of prompt attention:

I usually feel bad, I feel like picking a stone and hitting them. I usually leave crying saying I will never come back again. You may find them, and they just continue chatting and not listen to you, you tell them your issues and they don’t understand.

(FGD, Street-connected young women)

SCY also reflected upon experiencing a lack of patient-provider confidentiality, which may have far reaching consequences in the street community, as stated by one participant:

If we come here, some doctors should not disclose our secrets to anyone. They tell others as soon as I leave so there should be secrecy. If I come here sick and another child is told he will go to tell others on the streets and that will affect me.

(FGD, Street-connected young men)

However, SCY also exhibited some personal agency and recognized that not all providers will treat them the same way and one participant discussed making the decision to choose a different provider when unsatisfied with their interactions with a specific provider:

Some treat me well but if I go to other rooms, they don’t treat me well, it depends on who I find there, if I get someone who isn’t good to me, I usually ask to see another doctor.

(Street-connected young woman)

## Discussion

Our findings indicate a lack of health system responsiveness to SCY, particularly with respect to the elements of trust, dignity, prompt attention, choice of provider, and in some cases, confidentiality and quality of basic amenities. Our analysis suggests that this lack of health system responsiveness was driven by contextual determinants, particularly economic and socio-cultural ones, and negative patient-provider interactions. We found that these interactions were shaped by provider perceptions of, and emotions about, SCY. These findings offer valuable insight into key issues that need to be addressed in the health system in Kenya in order to strengthen health system responsiveness for SCY and possibly other marginalized and underserved populations.

Economic factors, such a lack of money and out-of-pocket cost for healthcare, were the most common contextual determinant that shaped SCY’s interactions with the health system. The inability to pay for care led to SCY reporting experiencing an absence in choice of healthcare provider, a lack of prompt attention, and indignity. In addition, the economic context and resource-constraints in Kenya likely impact the ability to of the health system to respond to patient expectations with respect to the quality of basic amenities, such as sufficient healthy food while hospitalized [29]. These findings underscore the need to enhance efforts towards universal health coverage and increased public expenditure on the health system, which is currently limited [26]. As Malhotra and Kyung Do (2017) found in their analysis of public health expenditure across 63 countries, a higher proportion of public health expenditure over total health expenditure was associated with health systems’ responsiveness for the poorest individuals, regardless of the country’s GDP [27]. Universal health coverage, ideally, will result in a health system that allows individuals to equitably receive healthcare. For SCY, universal health coverage would likely alleviate issues related to adverse experiences in relation to ‘choice of provider’ and ‘prompt attention’ that were linked to an inability to pay. Intertwined with an inability to pay, were experiences of indignity, that also intersected with SCY’s stigmatized social identity.

The experience of dignity in the context of health systems’ responsiveness requires that individuals seeking care are treated as persons rather than merely as patients with limited knowledge. A dignified experience is one where the individual’s human rights are safeguarded, they are treated respectfully by healthcare providers, have the right to ask questions and be provided information, and are afforded privacy during examination and treatment [29]. Our analysis reflects that SCY discussed that they were not treated respectfully in their interactions with providers nor as people in their own right. This is likely due to their stigmatized social identity as ‘*chokoraa*’ that associates this marginalized group with stereotypes of being violent, thieves, and ‘bad children’, which results in them being ‘othered’ and viewed as ‘not quite human’, whereby they then lose status in society and experience significant discrimination [21]. This socially and culturally constructed stigmatization of SCY shaped their interactions with providers and the health system, resulting in them not being treated with dignity as persons. In spite of this, SCY demonstrated some agency to the extent they were able, for example in trying to find another provider who would treat them better. Patient-centered care approaches and addressing stigma will be critical to meeting the needs of SCY in the healthcare system going forward [36].

Stigma is a barrier to accessing healthcare among other marginalized groups and young people in sub-Saharan Africa [37, 38]. Several approaches to reducing stigma in the health system with respect to specific diseases and mental health have been reported [39–42]. These approaches have included the provision of information, interventions for clinical practice, skills building activities, participatory learning, contact with the stigmatized group, empowerment approaches, and structural and policy changes [39, 40]. Limited evidence-based interventions exist to reduce stigma in the health system and few have been tested in low- and middle-income countries [39, 40, 42]. Building on this limited evidence, there is a need to rigorously develop and test interventions to reduce stigma in the health system in low- and middle-income countries, particularly for marginalized and vulnerable young people. Our analysis suggests that patient-provider interactions were heavily influenced by provider perceptions and emotions with respect to SCY as a result of stigma. Reducing stigma may lead to SCY experiencing more dignity and prompt attention in their interactions with the health system and providers.

SCY and healthcare providers predominantly reported negative patient-provider interactions. Our analysis suggests that these interactions were heavily influenced by providers’ stigmatized perceptions of and emotions about SCY. When exploring what drove these negative interactions, our analysis suggests that negative emotions (fear, pity, ambivalence, etc.) arising from preconceived perceptions and stigmatized characteristics of SCY by healthcare providers, influenced these undesirable patient-provider interactions. An important finding with respect to providers was the dichotomy between providers’ acknowledgement of their professional obligations to provide care and an individual’s ‘right to health’ and their apprehension about interacting with SCY. This ambivalence was coupled with an absence of expressing compassion (“the feeling or emotion, when a person is moved by the suffering or distress of another, and by the desire to relieve it” [43]) and empathy towards SCY, and predominantly expressing fear, pity (“a feeling of sadness or sympathy for someone else’s unhappiness or difficult situation” [44]), and sympathy. Yet, compassion is the foundation of quality healthcare, and is linked with dignity and human rights in medicine [45]. Compassion requires a profound awareness of a person’s suffering combined with the desire to alleviate it. It has been debated whether compassion is an innate quality or can be developed or nurtured in healthcare providers through specific and on-going training [45]. While teaching compassion may be difficult and there lacks any clear evidence-based approaches to nurturing compassion, some interventions exist to develop compassion using experiential and reflective learning focused on personal and professional experiences [45]. Culturally appropriate interventions could be developed or adapted and used for on-going clinical training with providers in Kenya in conjunction with interventions to reduce stigma, with the goal of increasing awareness about SCY’s predicament, cultivating a desire to respond to their situation and alleviate suffering, while reducing the stigmatizing perceptions and attitudes held by providers.

This study has limitations. First, this sub-analysis was conducted as part of a larger qualitative study that sought to understand community perceptions of SCY and wasn’t solely focused on the concept of health systems responsiveness. Interviews were not guided explicitly with respect to the concept of health systems responsiveness or the elements of Mirzoev & Kane’s framework and therefore our data may not have fully captured all aspects of health systems responsiveness in this context. Second, we were only able to interview healthcare providers in one county working in a national referral hospital, and therefore we did not elicit the views and experiences of healthcare providers across other counties and tiers of health facilities, so our findings may not be generalizable across Kenya’s health system. However, we were able to describe SCY’s experiences across counties, which were broadly in agreement, potentially signifying that patient-provider interactions and provider responses are similar in other counties. Third, these findings may not be generalizable to understanding SCY’s experiences of health system responsiveness in other low- and middle-income countries given the importance of contextual-level determinants in shaping the interactions between SCY, the health system, and providers. Despite these limitations, our analysis has many strengths. Our study explicitly asked about patient-provider interactions and SCY’s experiences with healthcare providers and the health system and included the perspectives of a broad range of providers including clinicians, nurses, social workers, HIV testing counsellors, and peer navigators. Our study also captured the contextual-level determinants of health system responsiveness which have been often overlooked in frameworks. Our analysis shed light on how these contextual factors influenced patient-provider interactions and importantly revealed that provider emotional responses may be a significant element of consideration in health systems responsiveness, which is a novel finding.

## Conclusions

This study suggests that the health system is inadequately responsive to SCY in Kenya. Increasing public health expenditure and expanding universal health coverage may begin to address economic factors, such as the inability to pay for care, which influence SCY’s experiences of choice of provider, prompt attention, and dignity. The deeply embedded adverse emotional responses expressed by providers about SCY, associated with the socially constructed stigmatization of this population, needs to be addressed to improve patient-provider interactions. A focus on nurturing compassion in healthcare providers in combination with reducing stigma in the health system, may improve SCY’s experiences of healthcare.

## Supplementary Information

**Additional file 1.**

## Data Availability

The datasets used and/or analysed during the current study are available from the corresponding author on reasonable request.

## Abbreviations

SCY: Street-connected children and youth
WHO: World Health Organization
IDIs: In-depth interviews
FGDs: Focus group discussions
AMPATH: Academic Model Providing Access to Healthcare
MTRH: Moi Teaching and Referral Hospital
CBOs: Community-based organizations

## Footnotes

1 In some public hospital facilities, SCY receive a waiver for care, and therefore do not pay for services. However, the system of waivers is inconsistent across county healthcare facilities and not all SCY are aware of how to navigate the system to receive a waiver.
